# Comparisons of maximum deformation and failure forces at the implant–abutment interface of titanium implants between titanium-alloy and zirconia abutments with two levels of marginal bone loss

**DOI:** 10.1186/1475-925X-12-45

**Published:** 2013-05-20

**Authors:** Chiung-Fang Wang, Heng-Li Huang, Dan-Jae Lin, Yen-Wen Shen, Lih-Jyh Fuh, Jui-Ting Hsu

**Affiliations:** 1 School of Dentistry, College of Medicine, China Medical University, Taichung 404, Taiwan; 2 Department of Dentistry, China Medical University and Hospital, Taichung 404, Taiwan; 3 Department of Dental Hygiene, College of Health Care, China Medical University, Taichung 404, Taiwan

**Keywords:** Abutment, Dental implant, Failure force, Maximum deformation force, Titanium alloy, Zirconia

## Abstract

**Background:**

Zirconia materials are known for their optimal aesthetics, but they are brittle, and concerns remain about whether their mechanical properties are sufficient for withstanding the forces exerted in the oral cavity. Therefore, this study compared the maximum deformation and failure forces of titanium implants between titanium-alloy and zirconia abutments under oblique compressive forces in the presence of two levels of marginal bone loss.

**Methods:**

Twenty implants were divided into Groups A and B, with simulated bone losses of 3.0 and 1.5 mm, respectively. Groups A and B were also each divided into two subgroups with five implants each: (1) titanium implants connected to titanium-alloy abutments and (2) titanium implants connected to zirconia abutments. The maximum deformation and failure forces of each sample was determined using a universal testing machine. The data were analyzed using the nonparametric Mann–Whitney test.

**Results:**

The mean maximum deformation and failure forces obtained the subgroups were as follows: A1 (simulated bone loss of 3.0 mm, titanium-alloy abutment) = 540.6 N and 656.9 N, respectively; A2 (simulated bone loss of 3.0 mm, zirconia abutment) = 531.8 N and 852.7 N; B1 (simulated bone loss of 1.5 mm, titanium-alloy abutment) = 1070.9 N and 1260.2 N; and B2 (simulated bone loss of 1.5 mm, zirconia abutment) = 907.3 N and 1182.8 N. The maximum deformation force differed significantly between Groups B1 and B2 but not between Groups A1 and A2. The failure force did not differ between Groups A1 and A2 or between Groups B1 and B2. The maximum deformation and failure forces differed significantly between Groups A1 and B1 and between Groups A2 and B2.

**Conclusions:**

Based on this experimental study, the maximum deformation and failure forces are lower for implants with a marginal bone loss of 3.0 mm than of 1.5 mm. Zirconia abutments can withstand physiological occlusal forces applied in the anterior region.

## Background

Treatments for missing teeth include fixed bridges, removable partial dentures, and dental implants [1-5]. The survival rates of dental implants are generally high. Wennerberg and Albrektsson reported that implants are successfully placed in 96–97% of prosthetic constructions, and that 87–97% of such implants are still in use after 5 years [6]. Numerous factors affect the success rate of implants [7]. Goodacre et al. divided the causes of implant failure into the following six categories: surgery, implant loss, bone loss, peri-implant soft tissue, mechanical difficulties, and esthetic/phonetic problems [8]. Esthetic problems are a challenge for dentists. Titanium abutments respond favorably to gum tissue and have favorable mechanical properties. However, the unesthetic bluish color of titanium remains visible through the soft tissues [9-11], which is of great concern when the maxillary incisors are treated, especially in patients with a high smile line or a thin mucosal biotype. The increasing demand for more favorable esthetic outcomes makes ceramic a potentially attractive alternative material because of its toothlike color.

The first ceramic abutments were developed in the 1990s to mitigate the unsightly color of titanium. The initial replacements were made of alumina [12,13]; however, several clinical studies [14,15] found fractures on alumina abutments. This weakness prompted the development of yttrium-oxide-stabilized zirconia, which is now widely used in dentistry [16]. Vagkopoulou et al. demonstrated that the transformation-toughening characteristic of yttrium-oxide-stabilized zirconia is effective at preventing crack propagation [16]. Yildirim et al. [17] indicated that yttrium-oxide-stabilized zirconia ceramic has twice the strength of alumina ceramic. However, whether zirconia abutments will entirely replace titanium abutments remains unclear.

In addition to esthetic problems, bone loss is another common concern in implant failures. Goodacre et al. [8] observed a mean bone loss of 0.9 mm (range from 0.4 to 1.6 mm) after 1 year, with an ongoing annual bone loss of 0.1 mm (range from 0 to 0.2 mm). Roos-Jansåker and colleagues [18] found that the bone level was located 3 mm apical to the implant head in 20.4% of Brånemark implants after 9 to 14 years of use. They further claimed that 8% of the implants suffered from progressive bone loss (≥1.8 mm) after 9 to 14 years when compared with 1-year data. From 1997–2004, 3609 implants were reported as failures, for reasons including implant mobility, inflammation/bone loss, and breakage [19].

While titanium-alloy and zirconia abutments are themselves resistant to breakage, peri-implant marginal bone loss can result in implant failure. These two materials have distinct mechanical properties, but few studies have compared them in detail. Therefore, the objectives of this study were to determine the maximum deformation and failure forces of titanium implants with titanium-alloy abutments and zirconia abutments in the presence of 3.0 and 1.5 mm of marginal bone loss. In addition, damage to the implant–abutment interface was observed using scanning electron microscopy (SEM).

## Methods

### Sample preparation

**Figure 1:**
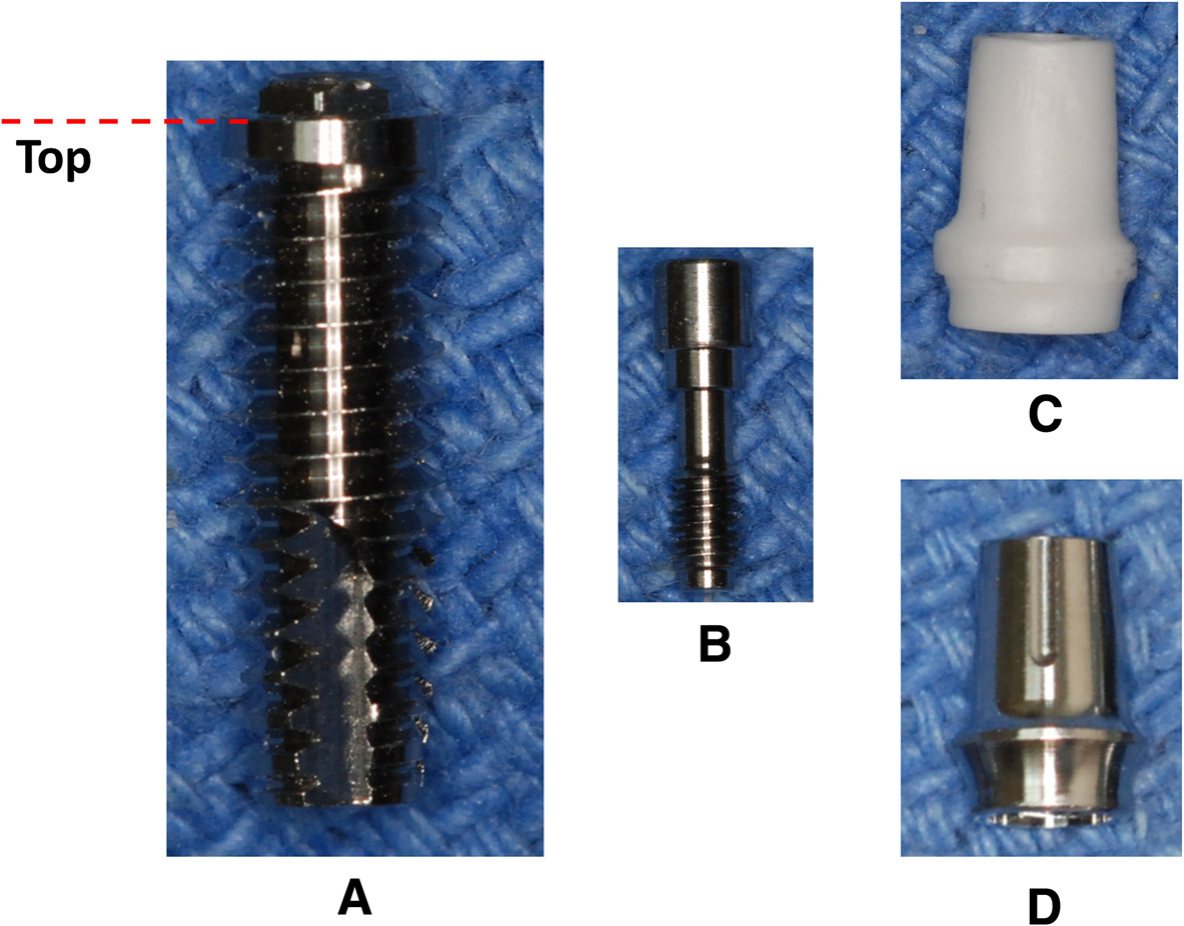
**Components of the type of dental implant used in this study.** (**A**) titanium implant, (**B**) titanium-alloy abutment screw, (**C**) zirconia abutment, and (**D**) titanium-alloy abutment. The top point of the implant is also shown in the Figure (**A**).

Twenty regular titanium implants (MK III, Nobel Biocare) were used in this study. Each implant was 4.0 mm in diameter and 13.0 mm in length, with a 0.7-mm-high external hexagon (Figure 1). Two abutment materials—titanium alloy (Grade 5) and zirconia (commercially unavailable)—were used in this experiment. All of the experimental abutments were 4.0 mm wide and 7.0 mm long. Each abutment was retained with a titanium-alloy (Grade 5) abutment screw tightened to 35 N · cm.

### Maximum deformation and failure forces of the combined implant and abutment

**Figure 2:**
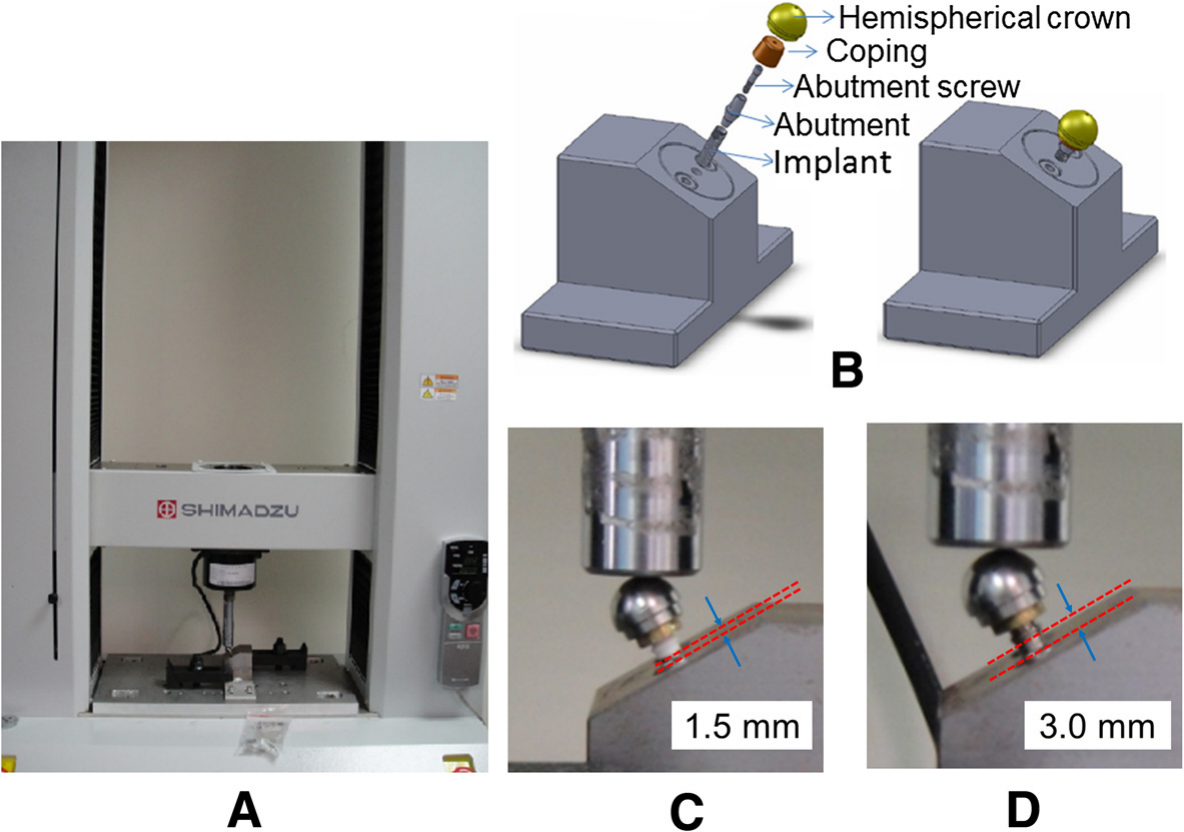
**Static loading of an implant–abutment component.** (**A**) overview, (**B**) exploded and assembled diagrams of the test setup, (**C**) close-up view of Group B2 (simulated bone loss of 1.5 mm with a zirconia abutment), and (**D**) close-up view of Group A1 (simulated bone loss of 3.0 mm with a titanium-alloy abutment).

The specimens were arranged in a modified version of the setup specified for the ISO 14801 standard (dentistry-fatigue test for endosseous dental implants, International Organization for Standardization 2007, Geneva, Switzerland) (Figure 2). Marginal bone loss after implantation was mimicked in Groups A and B by locating the top of the implants at 3.0 and 1.5 mm from the specimen holder, respectively. Furthermore, Groups A and B were each divided into two subgroups with five implants each: (1) titanium implants connected to titanium-alloy abutments (Groups A1 and B1 with simulated bone losses of 3.0 and 1.5 mm, respectively) and (2) titanium implants connected to zirconia abutments (Groups A2 and B2 with simulated bone losses of 3.0 and 1.5 mm, respectively).

**Figure 3:**
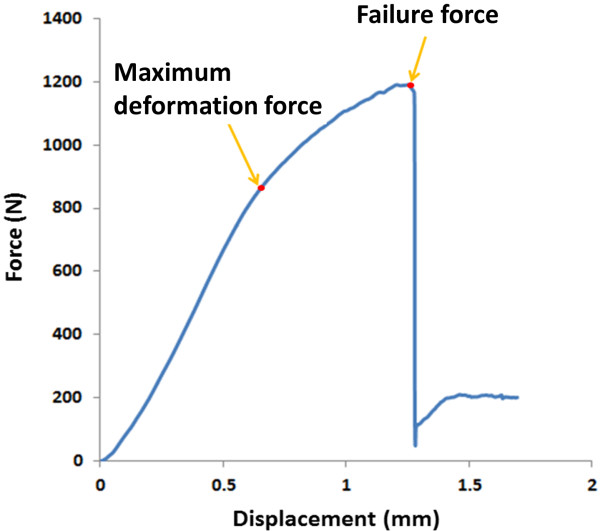
Force-vs-displacement curves for Group B2 (test #5) (simulated bone loss of 1.5 mm with a zirconia abutment).

Each implant–abutment assembly was embedded in a custom-made jig that allowed a 30-degree oblique force to be applied in the experiments based on the ISO 14801 standard. In accordance with this standard, a custom-designed hemispherical stainless-steel cap (with a diameter of 20 mm) was placed on the abutment instead of the prosthetic crown in this experiment. Each loading procedure involved applying a force to a hemispherical stainless-steel cap until failure, using a universal testing machine (AG-IC, Shimadzu, Japan) with a cross-head speed of 1.0 mm/min. The raw data were recorded as a force-vs-displacement curve. The first obvious turning point of the curve was defined as the maximum deformation force, and the top of the curve was defined as the failure force (Figure 3).

### Observing damage to the implant–abutment interface

SEM (JEOL, JSM-5400, Japan) was used to observe interfacial damage between the titanium implant and the abutment performed before and after mechanical testing at 35× magnification. For these evaluations the implants and abutments from all groups were cleaned with alcohol and dried, and the surfaces of the zirconia abutments were then coated with gold.

### Statistical analysis

The maximum deformation and failure forces obtained from the force-vs-displacement data of implants with titanium-alloy and zirconia abutments with exposures of 3.0 and 1.5 mm were summarized as mean and SD (standard deviation) values. Due to the small sample, the nonparametric Mann–Whitney test was used to analyze the forces at maximum deformation and failure, and to assess differences according to the exposure distance. The level of significance was determined as *P* < 0.05. All statistical analyses were performed using SAS software (Version 9.1.2, SAS Institute, Cary, NC, USA).

## Results

Table 1 indicates that the maximum deformation forces in all groups ranged from 450 to 1200 N, and that the failure force ranged from 550 to 1500 N. In simulating a peri-implant bone loss of 3.0 mm, the mean maximum deformation forces in Groups A1 and A2 were 540.6 and 531.8 N, respectively; the mean failure forces were 656.9 and 852.7 N, respectively. The use of titanium-alloy or zirconia abutments did not influence either the maximum deformation force (*P* = 0.62) or the failure force (*P* = 0.06). In simulating a peri-implant bone loss of 1.5 mm, the mean maximum deformation forces in Groups B1 and B2 were 1070.9 and 907.3 N, respectively; the mean failure forces were 1260.2 and 1182.8 N, respectively. The failure force did not differ significantly between Groups B1 and B2 (*P* = 0.41), whereas the maximum deformation force was higher in Group B1 than in Group B2 (*P* = 0.04), being larger for titanium-alloy abutments than for zirconia abutments. In all cases the implants were permanently bent and cracked in the region of their threads. No deformation of the abutment screws was observed in Group A1 or A2 (simulating 3 mm of bone loss). However, the abutment screw was fractured in two samples of Group B1 and in one sample of Group B2 (both simulating 1.5 mm of bone loss), while the others were only bent. The maximum deformation and failure forces differed significantly between 3.0 and 1.5 mm of bone loss in both titanium-alloy abutments (Groups A1 and B1, respectively; *P* < < 0.05) and zirconia abutments (Groups A2 and B2, respectively; *P* < < 0.05).

**Table 1:** Measured maximum deformation and failure forces in newtons (N) for the different groups

		**Bone loss of 3.0 mm**		**Bone loss of 1.5 mm**	
		**Group A1**	**Group A2**	**Group B1**	**Group B2**
		**Titanium- alloy abutment**	**Zirconia abutment**	**Titanium- alloy abutment**	**Zirconia abutment**
Maximum deformation Failure force (N)	Minimum	528.8	487.3	936.9	779.3
	Maximum	562.3	573.5	1182.6	1035.6
	Mean	540.6	531.8	1070.9	907.3
	SD	13.9	35.4	81.2	105.9
**Failure** force (N)	Minimum	553.4	714.0	1150.3	989.9
	Maximum	941.9	989.4	1385.5	1424.3
	Mean	656.9	852.7	1260.2	1182.8
	SD	161.5	116.9	94.2	148.9

**Figure 4:**
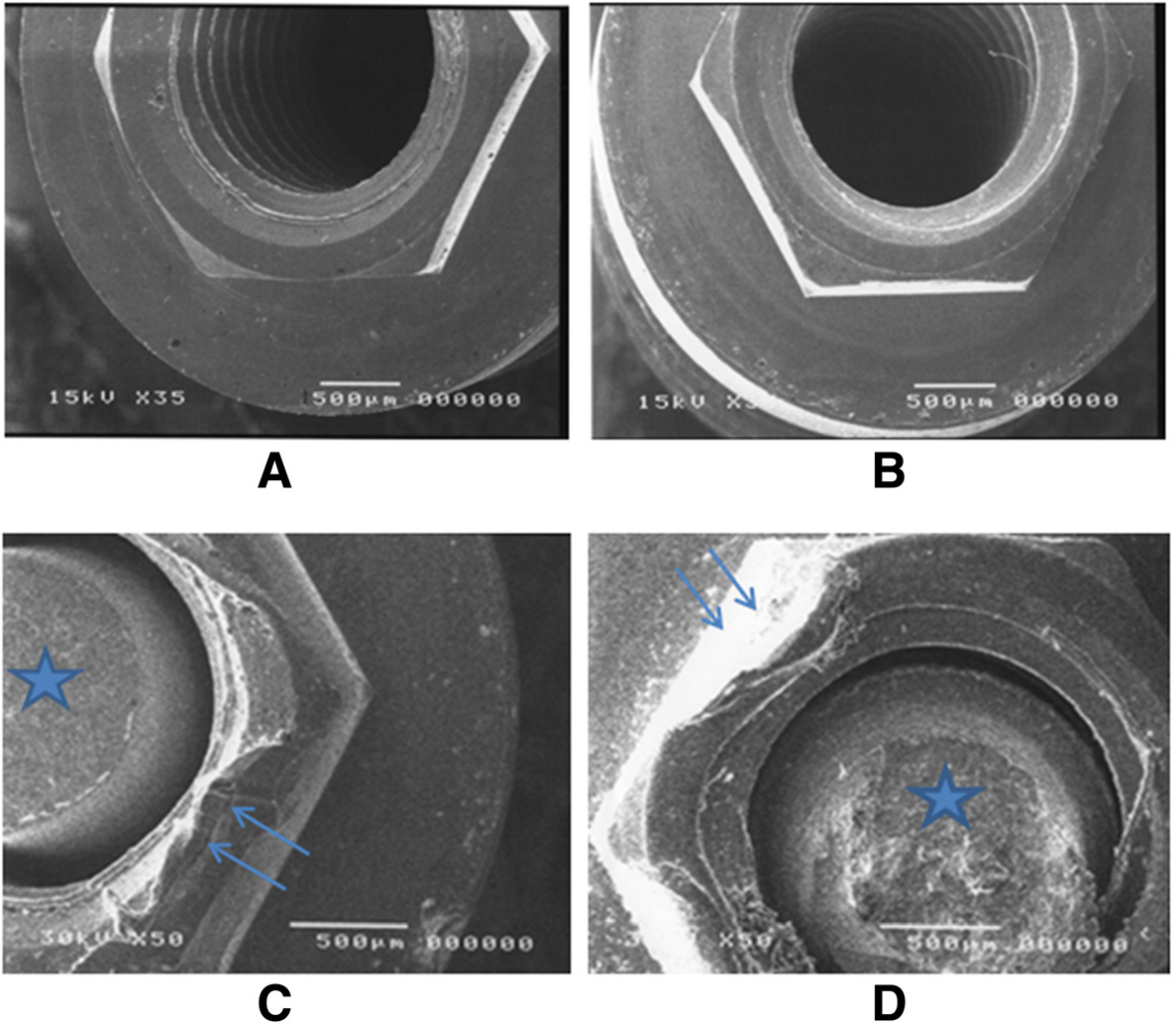
**SEM of implant surfaces with exposure distances of 3.0 and 1.5 mm.** (**A**) titanium implant in Group A1, (**B**) titanium implant in Group A2, (**C**) titanium implant with failed titanium-alloy abutment screw in Group B1, and (**D**) titanium implant with failed titanium-alloy abutment screw in Group B2. Stars indicate failed abutment screws, and arrows point to the damaged external hexagon of the implant.

SEM performed after the mechanical testing revealed no clear difference in damage to the implant–abutment interface between Groups A1 and A2 (simulating 3 mm of bone loss) (Figure 4A, B), with this damage categorized as minor. The surface deformation of the abutment or implant was more serious for a bone loss of 3.0 mm (Groups B1 and B2), with this damage being serious and permanent in both groups (Figure 4C, D).

## Discussion

Several advantages of zirconia have prompted its use by increasing numbers of dentists and patients: its strength is similar to that of other metals, its color and translucency are identical to those of teeth, and it exhibits high biocompatibility, which reduces the risk of inflammation because dental plaque is unlikely to accumulate. However, the strength of zirconia abutments is not as well understood as that of titanium abutments. Some studies have investigated the strength of zirconia abutments, but none of them considered the limit strength and interface damage mode of zirconia abutments that can cause a peri-implant bone to fail over time. Therefore, the main purpose of this study was to determine the maximum deformation and failure forces of titanium implants with titanium-alloy and zirconia abutments under different simulated bone-loss conditions.

Many clinical failure modes has been reported for dental implants [20,21]. In addition to infection soon after dental implant insertion and poor oral hygiene, another major reason for such failures is poor osseointegration, which refers to the interface condition between the implant and the surrounding bone. Osseointegration may be poor due to overloading [22] or primary instability [2,23,24] of the implant. Some marginal bone loss is generally observed after dental implant insertion, which begins at the neck of the implant and can spread to the first thread of the implant body or to the first contact between the bone and the rough surface of the implant. Sunden Piknér and Gröndahl [25] reported that 2.3% of implants demonstrated a marginal bone loss ≥3 mm within the first year, with this proportion increasing to 7.0% after 9 years. Rocci et al. [26] observed 97 implants with turned surfaces in 46 maxillae that were used for single or partial rehabilitation, and found an average surrounding bone loss of 1.5 mm after 3 years of prosthetic loading. Various factors such as the implant design in the cervical region, abutment design, biologic width, and platform-switching concept influence bone loss [27], and the reported range of marginal bone loss in the first year is 0.4 to 1.6 mm, with an annual rate of 0 to 0.2 mm [8] thereafter. The experiments performed in the present study exposed the top 3.0 and 1.5 mm of the implant, which was designed to simulate the typical clinical conditions after 5–7 and 20 years, respectively [8], thereby examining the influence of implant marginal bone loss on both medium- and long-term bases.

Yildirim et al. [17] used static tests to compare the fracture load of zirconia and aluminum-oxide abutments to 30-degree oblique loading, and found that the zirconia abutments (737.6 N) were stronger than aluminum-oxide abutments (280.1 N). Kerstein et al. [28] applied 40-degree oblique loading to measure the fracture strength of two brands of zirconia abutment: an Atlantis abutment constructed from zirconia, and a Nobel Biocare Procera AllZirkon abutment. They found that the fracture strength was significant larger for the Atlantis abutment (831 N) than for the Nobel Biocare Procera AllZirkon abutment (740 N). However, for both types of zirconia abutments the failure load exceeded the maximum human bite force. The results of the mechanical tests of the strength of zirconia abutments in the present study may differ from those of the previous studies due to differences in the experimental setups, particularly the angle of the applied load, the implant and abutment sizes and shapes, and the design of the upper loading member. The experimental apparatus used in the present study was designed based on the IS0 14801 standard, with only the distance at the top of the exposed implant—which was the main research focus of this study—adjusted to allow comparisons.

Ferrario et al. [29] found that the single-tooth bite forces in healthy young male adults were 150 and 140 N for the central and lateral incisors, respectively. The physiological maximum incisor biting forces may up to 290 N depending on facial morphology and age [30]. The average maximum failure forces for all groups in the present study exceeded the mentioned bite forces in anterior areas, as well as for all specimens tested, for which plastic deformations first appeared under oblique compressive loads exceeding 480 N. Zirconia abutments can therefore be considered a valid alternative to titanium-alloy abutments in anterior areas. However, zirconia abutments must be further evaluated in the posterior area of the jaw bone because the occlusal force is substantially larger than that of the anterior teeth.

Calderon et al. [31] found that the maximum bite force varied from 656.1 to 108.9 N in females with bruxism (mean maximum bite force: 395.6 N), and from 999.3 to 262.8 N in males with bruxism (mean maximum bite force: 584.5 N). These results suggest that in patients with bruxism and serious marginal bone loss (>3.0 mm) surrounding the implant, both zirconia and titanium-alloy abutments should be applied with extreme care.

Applying oblique loading to a dental implant without marginal bone loss will result in stress concentration at the implant–abutment interface, which is the fulcrum (pivot) of the structure. The energy associated with the applied force would pass through the abutment and onto the implant–abutment interface and abutment screw, and finally to the bottom of the implant and surrounding host material. However, the energy associated with applying an oblique loading to an implant with marginal bone loss would pass through the abutment and onto the implant–abutment interface and abutment screw, to the exposed part (upper side) of the implant, and finally to the bottom of the implant and surrounding host material. In Groups A1 and A2 (with 3 mm of bone loss), the bottom of the abutment screw was located near the fulcrum, and thus the upper abutment screw could enhance the structural stiffness of the exposed part of the implant. The implant–abutment structure was weakest in the fulcrum, which was 3 mm below the top of the implant. Therefore, if the applied loading exceeded the strength of the implant, the implant would begin bending, the thread would then crack, and finally the implant would fracture. In Groups B1 and B2 (with 1.5 mm of bone loss), the lower part of the abutment screw was located in the unexposed part of the implant, which was embedded in the specimen holder. In addition, the upper (cylinder) portion of the implant had a larger structural stiffness than the thread of the implant. The stress would be concentrated at the implant–abutment interface, similar to the case without marginal bone loss. Therefore, most of the applied energy would act at the implant–abutment interface, resulting in serious damage.

Human occlusal forces are dynamic. However, a dynamic loading test was not used to simulate the aging process in this study, due to previous studies indicating that the aging process does not significantly affect the fracture strength of zirconia specimens [28,32,33]. Basically, the survivability of zirconia depends on its ability to withstand occlusal forces. The experimental results indicated that the maximum deformation forces in Groups A2 and B2 were 531.8 and 907.3 N, which are much smaller than normal human bite forces. In a very small number of clinical cases the patient’s bite force could be larger than these maximum deformation forces and the marginal bone loss could be larger than 3 mm, resulting in the failure of the implant–zirconia abutment component or fracture of the implant body, although the zirconia abutment itself should remain intact.

Some limitations of this study should be considered. First, the sample size was small, though this was also the case in previous studies [34,35]; future studies should investigate larger numbers of specimens. Second, this study focused on static loading rather than dynamic loading. Although previous studies [28,32,33] have indicated that the use of static or dynamic loading would have no significant affect on the strength of zirconia specimens, further fatigue experiments are needed to confirm this. Third, only a single type of implant was used in this study, and so the effect of different implant sizes and implant–abutment connection types should be also investigated in future studies.

## Conclusions

The experimental results obtained in single static loading tests indicated that implants with a simulated marginal bone loss of 3.0 mm exhibited decreased maximum deformation and failure forces compared to those with a simulated marginal bone loss of 1.5 mm. Zirconia abutments can withstand physiological occlusal forces applied in the anterior region for both 1.5-mm and 3.0-mm marginal bone losses. Therefore, the clinical use of zirconia abutments should be considered when esthetic outcomes are important.

## Competing interests

The authors declare that they have no competing interests.

## Authors’ contributions

CFW, JTH, and LJF conceived and designed the experiments; CFW, JTH, DJL, and YWS performed the experiments; HLH and JTH analyzed the data; CFW and JTH wrote the manuscript; and all of the authors read and approved the final version of manuscript.
